# 

**Published:** 1862-12

**Authors:** 


THE NEW SYDENHAM SOCIETY'S PUBLICATIONS.

*A Clinical Treatise on Diseases of the Liver; wood cuts and lithographs by* DR. FRIED. THEOD. FRERICHS, *Professor of Clinical Medicine in the University of Berlin, etc.; Medical Privy-Counsellor and Medical Adviser to the Ministry of Public Instruction and Medicine at Berlin, in two volumes, volume 2d. Translated by* CHARLES MURCHISON, M. D., F. R. C. P., *Physician to the London Fever Hospital, Lecturer on Pathological Anatomy, and Assistant Physician at the Middlesex Hospital. The New Sydenham Society, London,* 1861.

---

*A Year-Book of Medicine, Surgery, and their allied Sciences, for* 1861. *Edited by* DR. HARLEY, DR. HANDFIELD JONES, MR. HULKE, DR. GRAILY HEWITT, *and* DR. SANDERSON, *for the New Sydenham Society. London,* 1862.

We have received these books from the American Secretary, C. F. Heywood, 66 West 20th Street, New York City. They are exceedingly valuable works which we cannot attempt to review and give any idea of the contents or character in the space our Journal affords. Suffice it to say that the yearly publications of the Society are richly worth the yearly subscription of one guinea; indeed it is the best investment a physician can make who desires the most valuable books, and we advise all physicians who can afford the luxury to make subscription to the New Sydenham Society, which can be done through the American agent, Dr. C. F. Heywood, 66 West 20th Street, New York City.

The Society has just entered upon its fourth year. During the first three years it has issued thirteen works, comprising two Fasciculi of an Atlas of Skin Diseases, and eleven octavo volumes. It has been the especial aim of the Councll to select for the Society works of a practical character and sterling value, representing the opinions of the highest authorities of the present day. The works have been very varied as regards the departments of medieal science to which they relate.

The Society issues annually a *Year-book of Medicine and Surgery*, comprising reports in abstract of the more important contributions to professional literature of the year.

Two Fasciculi of an *Atlas of Skin Diseases* (life size) have already appeared, and the members will receive an additional one each year.

The Society already numbers about 3,500 members, but the Council trusts that when the great advantages which its offers becomes more widely known, a large accession may be yet obtained.

All the works for the three past years have been reprinted. and the whole series may be obtained on payment of three guineas.

The subscription is one guinea annually, to be paid in advance.

---

*The Hospital Steward's Manual, for the instruction of Hospital Stewards, Ward-Masters, and Attendants, in their several duties; prepared in strict accordance with existing regulations and the customs of service in the Armies of the United States of America, and rendered authoritative by order of the Surgeon-General; by* JOSEPH JANVIER WOODWARD, M. D., *Assistant Surgeon U. S. A., Member of the Academy of Natural Sciences of Philadelphia, etc. Philadelphia:* J. B. LIPPINCOTT & Co., 1862.

Every hospital steward in the United States Army, should either provide himself or be provided with this volume. It contains the minute detail of his whole duty, and will suggest to him many things highly proper for him to know, which will otherwise be likely to escape his observation. If possessed and carefully studied it will make its value apparent in the system, efficiency, intelligence and capacity with which a hospital steward will discharge the duties of his office. And we again repeat, no such officer should be without it, or ignorant of its contents.

---

*Anatomy of the Arteries of the Human Body, Descriptive and Surgical, with the Descriptive Anatomy of the Heart; by* JOHN HATCH POWER, M. D., *Fellow, and Member of Council, of the Royal College of Surgeons; Professor of Descriptive and Practical Anatomy in the Royal College of Surgeons; Surgeon to the City of Dublin Hospital, etc. Authorized and adopted by the Surgeon-General of the United States Army for use in Field and General Hospitals. Philadelphia:* J. B. LIPPINCOTT & Co., 1862.

The importance to the surgeon of a correct knowledge of the position and relation of the arteries, is too apparent to require comment; and a book which by precept and illustration enforces upon the mind these conditions will not fail of appreciation by intelligent surgeons. We have rarely, if ever, received a work of this character which so favorably impressed us, and we most earnestly recommend it to all who desire an accurate and definite understanding of the arteries and the operations which may be made upon them, as well as the mode of making such operations. Th illustrative drawings are truthful to nature, and exceedingly well executed; the parts are shown with the distinctness almost of actual dissection. For those physicians so situated as to be unable to resort at pleasure to the cadaver, and who are yet practicing surgery, to some extent at least, nothing can in so small compass exceed the value of this book. We have carefully read and compared it with actual dissection, and we find it plain, simple, truthful, complete, and altogether indispensable as a reference and guide.

### PAMPHLETS RECEIVED.

*The Atlantic for December.*—By Messrs. Ticknor & Fields, Boston.—The following is a list of the articles and the contributors:

The Procession of the Flowers, by T. W. Higginson; One of my Clients; The Cumberland, by H. W. Longfellow; The Fossil Man, by Charles L. Brace; Life in Open Air, by Theodore Winthrop; A Woman, by Rose Terry; About Warwick, by Nathaniel Hawthorne; Lyrics of the Street, by Julia Ward Howe; Mr. Axtell; My Hunt after "The Captain," by O. W. Holmes; Waiting.

*Peterson's Magazine.*—We are in receipt of this popular Lady's Magazine for December. It is a splendid number. The title page for 1863, containing portraits of the chief contributors, is very handsome. We are requested to give the following notice of it:

"'Peterson' will be greatly improved in 1863. It will contain 1000 pages of double column reading matter; 14 steel plates; 12 colored steel fashion plates; 12 colored patterns in Berlin work, embroidery or crotchet, and 900 wood engravings—*proportionately more than any other periodical gives.* Its stories and novelettes are by the best writers. In 1863, four Original Copyright Novelettes will be given. *Its Fashions are always the Latest and Prettiest!* Every neighborhood ought to make up a club.—Its price is but *two dollars* a year, or a dollar less than Magazines of its class. *It is the Magazine for the times!* To clubs, it is cheaper still, viz: three copies for $5, five for $7.50, or eight for $10. To every person getting up a club, the Publisher will send *an extra copy gratis, as a premium, or a large sized mezzotint for framing, "Bunyan Parting from his*

*Blind Child in Prison.*" Specimens sent (if written for) to those wishing to get up clubs."

Address, post-paid, Charles J. Peterson, 306 Chestnut street, Philadelphia:

---

*Catalogue of the Trustees, Overseers, Faculty and Students of the Berkshire Medical Institution, for the year* 1862, *and of the Graduates and Honorary Graduates, since its incorporation in* 1823. *Pittsfield, Mass., October*, 1862.

The Winter Reading Term of this Institution will commence on the first Wednesday of January, 1863, and continue sixteen weeks.

Thorough instruction will be given by Profs. Childs and Greene in the theoretical and practical branches of Medicine and Surgery.

---

*Godey's Lady's Book for December; published by* L. A. Godey, 323 *Chestnut Street, Philadelphia.*

This is one of the oldest and most deservedly popular magazines of the day. It comes to us beautifully illustrated, with a very interesting table of contents.

---

*Harper's New Monthly Magazine for December*—Table of Contents. Waiting for the Children; Poland Over-Ground and Under-Ground; A Withered Flower; Gas and Gas-Making; A Man's Life; The Stamp Act Congress; Love by Mishap; Roll-Call; Romola, by the author of "Adam Bede;" Random Recollections of a Life; Orley Farm, by Anthony Trollope; A Camp-Meeting in Tennessee; Mistress and Maid, a Household Story, by Miss Mulock; A Fairy in Search of a Place; The Small House at Allington; My First Sermon; Monthly Record of Current Events; Literary Notices; Editor's Easy Chair; Editor's Drawer; Fashions for December.

